# Caries detection enhancement using texture feature maps of intraoral radiographs

**DOI:** 10.1007/s11282-018-0354-8

**Published:** 2018-11-09

**Authors:** Rafał Obuchowicz, Karolina Nurzynska, Barbara Obuchowicz, Andrzej Urbanik, Adam Piórkowski

**Affiliations:** 1 Department of Diagnostic Imaging, Jagiellonian University Medical College, 19 Kopernika Street, 31-501 Cracow, Poland; 2 Institute of Informatics, Faculty of Automata Control, Electronics, and Computer Science, Silesian University of Technology, Akademicka 16, 44-100 Gliwice, Poland; 3 Department of Conservative Dentistry with Endodontics, Jagiellonian University Collegium Medicum, Montelupich 4, 31-155 Cracow, Poland; 4 Department of Geoinformatics and Applied Computer Science, AGH University of Science and Technology, Mickiewicza 30, 30-059 Cracow, Poland

**Keywords:** Digital intraoral radiography, Image preprocessing, Caries, Texture analysis

## Abstract

**Objectives:**

Dental caries are caused by tooth demineralization due to bacterial plaque formation. However, the resulting lesions are often discrete and thus barely recognizable in intraoral radiography images. Therefore, more advanced detection techniques are in great demand among dentists and radiographers. This study was performed to evaluate the performance of texture feature maps in the recognition of discrete demineralization related to caries plaque formation.

**Methods:**

Digital intraoral radiology image analysis protocols incorporating first-order features (FOF), co-occurrence matrices, gray tone difference matrices, run-length matrices (RLM), local binary patterns (LBP), and k-means clustering (CLU) were used to transform the digital intraoral radiology images of 10 patients with confirmed caries, which were retrospectively reviewed in a dental clinic. The performance of the resulting texture feature maps was compared with that of radiographic images by radiologists and dental specialists.

**Results:**

Significantly improved detection of caries spots was achieved by employing the CLU and FOF texture feature maps. The caries-affected area with sharp margins was well defined using the CLU approach. A pseudo-three-dimensional effect was observed in outlining the demineralization zones inside the cavity with the FOF 5 protocol. In contrast, the LBP and RLM techniques produced less satisfactory results with unsharp edges and less detailed depiction of the lesions.

**Conclusions:**

This study illustrated the applicability of texture feature maps to the recognition of demineralized spots on the tooth surface debilitated by caries and identified the best performing techniques.

## Introduction

Dental caries is a chronic infectious disease that is prevalent in approximately 44% of the worldwide general population under 20 years of age [1]. In the United States, approximately 90% of adults with symptoms of dental caries have this disease [2, 3]. Dental caries typically cause enamel loss due to contact with the surfaces of adjacent teeth [4]. The bacterial buildup (dental plaque formation) on the tooth surface and secondary lytic effect on the enamel caused by the acid formed as a byproduct of bacterial metabolism are considered the main factors responsible for enamel breakdown and the ultimate loss of enamel integrity [5, 6]. Detection of dental caries is usually performed by visual inspection. In particular, the examination of morphologically invisible lesions is based on intraoral radiographic studies [7].

Caries detection involves taking periapical, bitewing, and panoramic images, which have a sensitivity of less than 60% [8]. Panoramic images have the lowest sensitivity, whereas better results are achieved with bitewing and periapical radiographs. Therefore, the former techniques are preferable for dental caries imaging analysis [9–11]. Nevertheless, the methods of caries evaluation and qualification criteria remain the subjects of active discussion [12].

Determining the dimensions of subtle erosions on the internal side of the proximal part of the tooth is a difficult task. Moreover, the anatomical noise caused by the complexity of superimposed anatomical structures and multiple interpretations by imaging professionals often makes the diagnosis uncertain [13, 14]. Thus, it is not usually possible to detect dental caries in their initial stages and monitor their further progression [15]. Computer-based analysis of digital radiographic images of the tooth, which allows the user to vary the image characteristics with an exposition of shape margins and brightness levels on gray-scale blurred images, is, therefore, in great demand in the medical community.

This study was performed to develop an improved method for the clinical detection of dental caries using digital intraoral radiology (DIR) images.

### Related work

In 2015, a method called feature extraction, which allowed for computation of texture features from dental X-ray images, was proposed [16]. The intensity, mean, entropy, perimeter, and energy parameters of the selected region were calculated to determine whether the region of interest included caries. A three-layer auto-encoder was employed for feature calculation, while a softmax layer was used for classification. Despite the relatively high sensitivity (97%) of the proposed technique, the lack of information regarding the number of training and test data points as well as the detailed experimental procedure raised questions regarding whether this approach was sufficiently generalized to be applied to any input. A similar methodology involving deep neural networks, which enabled dentists to detect tooth decay on dental X-ray images, was subsequently proposed [17].

Prior to the above two studies, other researchers extracted multiple features from dental X-ray images using texture analysis techniques based on the gray level co-occurrence matrix (GLCM) [18]. Thus, contrast, correlation, entropy, homogeneity, and energy were computed to perform tooth segmentation. Even earlier, an automatic system for caries detection that transformed input images via the watershed segmentation of histograms was proposed [19]. Several GLCM features were subsequently derived and fed into a support vector machine classifier with a modified kernel function. The process of dental caries classification was also considered in a study in which the authors tested several classifiers, including artificial neural network classifiers, k-nearest neighbor classifiers, naive Bayes classifiers, and support vector machine classifiers, to develop a caries detection procedure [20].

Two particular studies reported in the literature were very similar to the present study [21, 22]. The authors proposed a simple transformation aimed at caries detection, after which GLCM-based texture analysis was performed to identify cysts and caries-affected areas. The GLCM texture features, including energy, entropy, homogeneity, contrast, and correlation, were used for segmentation of cyst regions.

Texture feature analysis was also applied to evaluation of the bone structure affected by osteoporosis [23–25], and similar techniques were used to characterize periapical bone loss [26–29], periapical bone healing [30–33], and focal periapical lesions [34, 35]. Other research has focused on evaluation of the jaws [36] and cases in which sex-dependent structural differences in the jaw bone are visible [37].

## Materials and methods

### Data collection

This retrospective work involved the use of 10 anonymized images (periapical radiographs) of different patients admitted to our dental clinic in 2017. The areas containing suspected lesions were analyzed. All radiographs were obtained under identical conditions.

**Fig. 1:**
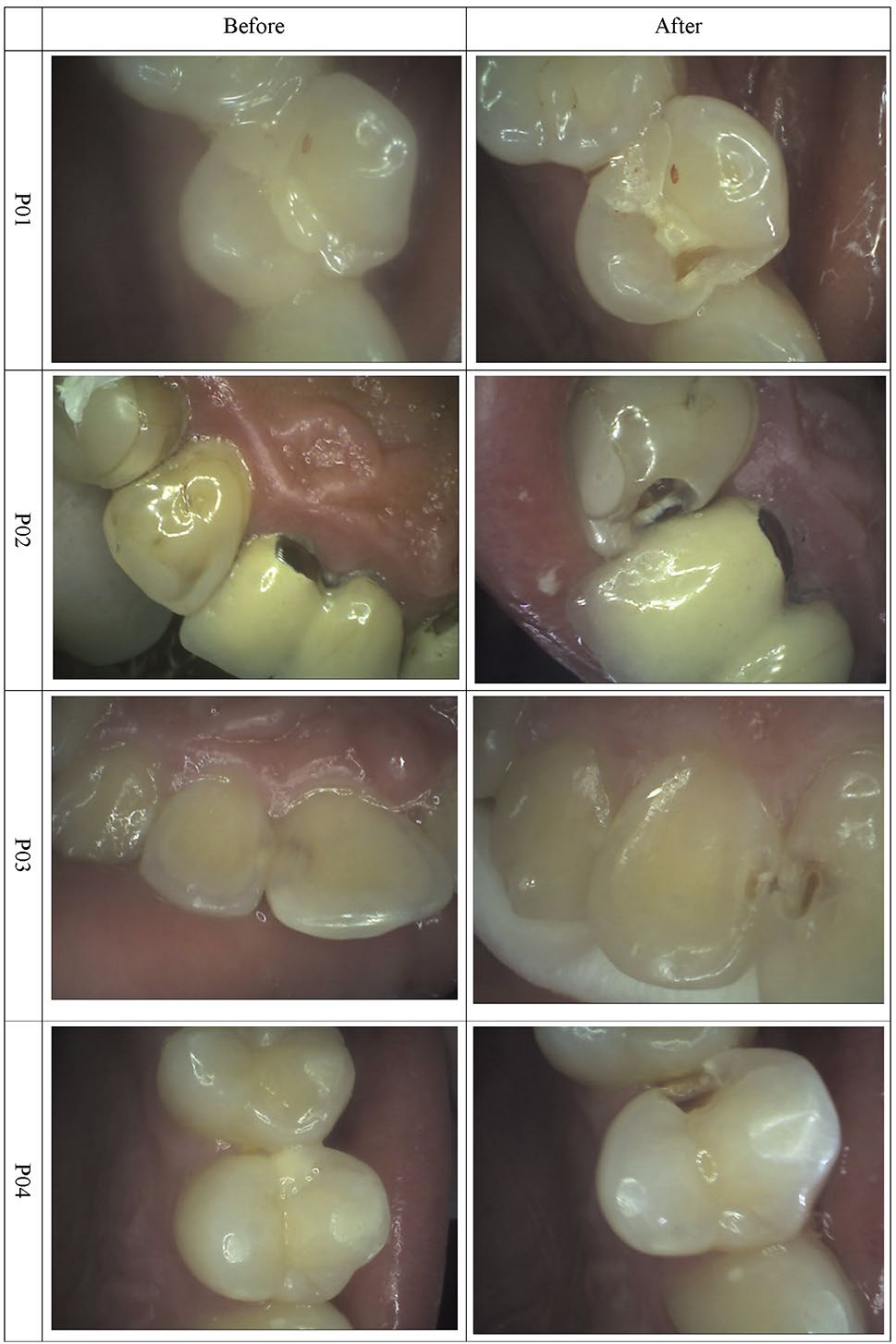
Photographs captured with intraoral digital camera before and after cavity preparation

The patients’ charts included cases of clinically (intraoperatively) confirmed superficial and medium caries as well as one case of deep caries taken from a local picture archiving and communication system, all of which were retrospectively studied. The inclusion criteria were limited visibility in the entry DIR images and the presence of photographic intraoral documentation (Fig. 1).

The texture feature maps were analyzed independently by two radiologists with experience in reading dental radiographs. The interobserver correlation between the readers was determined. The following features of changes in the radiographic images were examined: the tissue contrast with a special focus on the caries-affected area, the content of the evaluated shape, the caries outlines, and the sharpness of the margins. The obtained radiological results were verified by a dental specialist involved in the treatment of the evaluated cases.

The DIR images were obtained using a dental X-ray system (Kodak CS 6500; Carestream Dental, Atlanta, GA, USA) at an image quality of 70 kVp, current of 7 mA, mean exposure time of 0.05 s, pixel spacing of 0.018 mm, and 12-bit resolution corresponding to the radiodensity. A Digital Imaging and Communications in Medicine lossless system was used for file storage.

### Methods

The computer vision domain provides many powerful tools for the recognition of image content, including an approach based on image texture analysis, which allows segmentation of regions of interest by statistical analysis of the image pixel neighborhood. Additionally, clustering or quantization methods can be utilized to automatically merge regions of a given signal range by additionally taking their spatial positions into account.

In the present study, texture feature maps were generated by several methods. This approach significantly differs from the one-feature-per-image calculations widely used in the literature [38, 39]. Instead of computing a single feature describing the entire image, feature values were calculated for each pixel position. For this purpose, a moving window with a reasonably smaller size than the image resolution is typically selected. In our simulations, a square window with a resolution of 21 × 21 pixels was employed. The window was centered over the pixel of interest and then moved along the image coordinates to determine the feature values for all pixels in the input image. Most procedures described below were performed in this manner to produce feature maps with a number equal to the number of parameters. All proposed methods were implemented in the Matlab® 2016 environment (MathWorks, Natick, MA, USA).

### First-order features

An image histogram describes the probability of detection of a particular intensity within the data, and its shape can be used to determine important image parameters including sharpness, contrast, and number of objects. First-order features (FOF) are a formalized representation of such information. For image I with spatial resolution W × H and intensity range G, the normalized histogram is defined by the following equation:

$$H\left( i \right)=\frac{1}{{WH}}\mathop \sum \limits_{{x=1}}^{W} \mathop \sum \limits_{{y=1}}^{H} \left\{ {\begin{array}{*{20}{c}} 1&{I\left( {x,y} \right)=i} \\ 0&{{\text{otherwise}}} \end{array}} \right\}~~~~~~i~\epsilon \left[ {0..G - 1} \right]$$

This equation can be used to derive six different features (mean, variance, skewness, kurtosis, energy, and entropy) as follows:

$${\text{FO}}{{\text{F}}_{{\text{mean}}}}=\frac{1}{{WH}}\mathop \sum \limits_{{x=1}}^{W} \mathop \sum \limits_{{y=1}}^{H} I\left( {x,y} \right)$$

$${\text{FO}}{{\text{F}}_{{\text{variance}}}}=\frac{1}{{WH}}\mathop \sum \limits_{{x=1}}^{W} \mathop \sum \limits_{{y=1}}^{H} {\left( {I\left( {x,y} \right) - {\text{FO}}{{\text{F}}_{{\text{mean}}}}} \right)^2}$$

$${\text{FO}}{{\text{F}}_{{\text{skewness}}}}=\frac{1}{{WH}}\mathop \sum \limits_{{x=1}}^{W} \mathop \sum \limits_{{y=1}}^{H} {\left( {I\left( {x,y} \right) - {\text{FO}}{{\text{F}}_{{\text{mean}}}}} \right)^3}{\left( {\sqrt[2]{{{\text{FO}}{{\text{F}}_{{\text{var}}}}}}} \right)^{ - 3}}$$

$${\text{FO}}{{\text{F}}_{{\text{kurtosis}}}}=\frac{1}{{WH}}\mathop \sum \limits_{{x=1}}^{W} \mathop \sum \limits_{{y=1}}^{H} \left\{ {{{\left( {I\left( {x,y} \right) - {\text{FO}}{{\text{F}}_{{\text{mean}}}}} \right)}^4}{{\left( {\sqrt[2]{{{\text{FO}}{{\text{F}}_{{\text{var}}}}}}} \right)}^{ - 4}}} \right\} - 3$$

$${\text{FO}}{{\text{F}}_{{\text{energy}}}}=\mathop \sum \limits_{{i=1}}^{G} H{\left( i \right)^2}$$

$${\text{FO}}{{\text{F}}_{{\text{entropy}}}}= - \mathop \sum \limits_{{i=1}}^{G} H\left( i \right){\text{~}}\log \left( {H\left( i \right)+\varepsilon } \right)$$

Here, the value of *ε* is relatively small to ensure that the logarithm function is not computed for 0. To obtain images for these features, their values were calculated by the moving window method with a spatial dimension of 21 × 21 pixels.

### Clustering

Another method that effectively highlights the image content is k-means clustering (CLU), which takes the pixel intensity into account. A previous study showed that the basic analysis of image intensities allows segmentation of medical objects [40]. In this technique, the number of search clusters must be defined (their initial values are initialized randomly). Each pixel of the image is then assigned to the nearest cluster using a distance metric (in this study, the Euclidean metric was selected). The new cluster value is set to the average value of the intensities of all its pixels, and the procedure is repeated until the cluster value becomes constant or the number of iterations reaches a specified threshold. Although this method demonstrates satisfactory performance, it also has several drawbacks. First, the number of clusters must be known *a priori*. Second, the colors used in the cluster images are assigned randomly. Finally, the random initialization results in different outcomes for the same input, and the method is computationally expensive due to its iterative nature.

Therefore, a special quantization procedure that can solve several clustering problems has been proposed. It divides all intensities of the image pixels into a given number of colors, but uses only the intensity information as a guide. While the previous method searches for the cluster center in the high-probability region of the histogram, equal spacing between colors is applied during quantization. As a result, the number of colors is identical to that in the previous technique, whereas the division varies slightly. The described method solves all of the above-mentioned problems except that the number of colors still must be set *a priori*.

### Gray level co-occurrence matrix

The GLCM [41] contains information on the spatial relations between the adjacent pixels in the texture. It is calculated as a matrix with entries that represent the probabilities of the coexistence of two gray tones next to each other. The distance between the analyzed pixels is used as a parameter (its value was set to 1 in this study). To eliminate the influence of texture rotation, it is reasonable to compute four different matrices at angles of 0°, 45°, 90°, and 135° in the adjacency direction and calculate their sum before feature calculations. Moreover, to facilitate the computation procedure, the input image is quantized to a lower number of gray levels to reduce the matrix size (in our experiments, the images were quantized down to 32 colors). It is possible to extract the following 14 features from this matrix: angular second moment, contrast, correlation, variance, homogeneity, sum average, sum variance, sum entropy, entropy, difference variance, difference entropy, two informational measures of correlation, and maximal correlation coefficient.

### Gray tone difference matrix

The texture described in the context of human texture perception is defined by the gray tone difference matrix (GTDM) [42]. Its column vector contains G entries, which represent the difference between the pixel intensity level *I* and the average illumination $\bar {I}$ in a small *K* × *K* neighborhood described as follows:

$${\text{GTDM}}\left( i \right)=~\mathop \sum \limits_{{x=1}}^{W} \mathop \sum \limits_{{y=1}}^{H} \left| {i - \bar {I}} \right|~~{\text{where}}~~\bar {I}=\frac{1}{{M - 1}}\mathop \sum \limits_{{m= - K}}^{K} \mathop \sum \limits_{{n= - K}}^{K} I\left( {x+m,y+n} \right),\left( {m,n} \right) \ne \left( {0,0} \right)$$

Here, *M* = (2*K* + 1)^2^. When a given illumination does not exist or the image has only one color, the corresponding entries are equal to zero. It is possible to derive five features from this matrix: coarseness, contrast, busyness, complexity, and strength.

### Run-length matrix

Another technique for texture characterization is the run-length matrix (RLM) method, which assumes that a texture of good quality is complex and can, therefore, be defined by rapid changes in illumination (pixel values), whereas the coarse texture is expressed by the larger sections of similar color [43]. This information is encoded in a matrix, the entries of which store the probabilities of the occurrence of a selected illumination value across a number of adjacent pixels. Because of the difficulty in defining the maximum length of the color run, it is acceptable to reduce the maximum length down to 10 and place all longer runs in the same matrix entry. Furthermore, for computational reasons (as in the case of GLCM), the gray levels in the image are quantized to a smaller number of intensities (32 in this study). To eliminate the rotation of input data, the matrix was computed at 0° and 90° and summed before feature calculation. In this study, 11 features were calculated [43–46], and their values are summarized in Table 1.

**Table 1:** Summary of texture feature maps computed using various techniques

Texture feature	GLCM	FOF	GTDM	RLM
F1	Angular second moment (energy)	Mean	Coarseness	Short run emphasis
F2	Contrast	Variance	Contrast	Long run emphasis
F3	Correlation	Skewness	Busyness	Gray level nonuniformity
F4	Variance	Kurtosis	Complexity	Run-length nonuniformity
F5	Inverse difference moment (homogeneity)	Energy	Texture strength	Run percentage
F6	Sum average	Entropy		Low gray level run emphasis
F7	Sum variance			High gray level run emphasis
F8	Sum entropy			Short run low gray level run emphasis
F9	Entropy			Short run high gray level run emphasis
F10	Difference variance			Long run low gray level run emphasis
F11	Difference entropy			Long run high gray level run emphasis
F12	Information measure of correlation I			
F13	Information measure of correlation II			
F14	Maximal correlation coefficient			

*GLCM* gray level co-occurrence matrix, *FOF* first-order features, *GTDM* gray tone difference matrix, *RLM* run-length matrix

### Local binary patterns

A completely different method for texture characterization is represented by local binary patterns (LBP) [47, 48]. This texture operator calculates a different representation of the input images as a straightforward result of its execution. For each input pixel I(x_c_,y_c_), a neighborhood defined by radius R and the number of evenly sampled points on the radius P is specified. The LBP function is calculated as follows:

$${\text{LB}}{{\text{P}}_{P,{\text{R}}}}\left( {{x_{\text{c}}},{y_{\text{c}}}} \right)=\mathop \sum \limits_{{p=0}}^{{P - 1}} s\left[ {I\left( {{x_{\text{c}}},{y_{\text{c}}}} \right) - I\left( {{x_p},{y_p}} \right)} \right] \cdot {2^p},$$

Here, the operator *s*[.] returns 1 for positive values and 0 otherwise.

### Laws’ texture energy measures

Another way to extract hidden information from the image is to use Laws’ texture energy measures (LAWS) [49]. This technique utilizes a predefined set of masks to calculate the local energy for a given image. These masks are designed to enable the detection of four texture characteristics, namely level, edge, spot, and ripple, which are then combined to describe more complex findings. In the present study, nine texture feature maps were derived using this technique.

### Preprocessing

Most of the proposed techniques are designed for 8-bit gray-scale images. Moreover, their implementation is performed at this data accuracy. Nevertheless, the processed data have a 12-bit precision; therefore, more information can be obtained using the entire scale. In previous studies, various methods were proposed to reduce the bit resolution and related noise level [50, 51]; however, the authors also considered the loss of information that occurred during the image resolution reduction from 12 to 8 bits and decided to examine whether an appropriate preprocessing method might improve the applicability of the proposed texture operator. Therefore, several transformations that highlighted certain ranges were considered. They were represented by histogram equalization (HEQ) and the statistical dominance algorithm (SDA) [52].

HEQ highlights larger areas with small illumination variations by increasing their contrast. Consequently, the tissue pattern becomes more noticeable, but some regions may be discriminated after the appearance of large objects. In this study, HEQ was performed both for the original 12-bit images (whose resolution was subsequently reduced to 8 bits) and for the images followed by method application. Although this technique is nondeterministic, its combination with other methods can create additional possibilities for data analysis. The order of performing the bit cut and HEQ procedures does not influence the generated texture feature maps.

The SDA represents another approach to the analysis of sensitive changes in image illumination. This technique is based on the equal treatment of mutual relations between pixels; nevertheless, their luminance values can be either very high or very low, which requires better visualization of the local texture pattern. The resulting SDA image is scaled to 8 bits, after which it is analyzed using a procedure similar to that used for the original image. As a result, all texture feature maps were calculated.

### Post-processing

The results obtained after application of all of the above-described texture analysis techniques may lead to different computer responses with various distributions. Therefore, the original images in this study were analyzed by applying a histogram stretching (HSTR) algorithm after 8-bit normalization and HEQ procedure followed by scaling the results to the 8-bit resolution.

### Summary of texture feature map analysis techniques

**Fig. 2:**
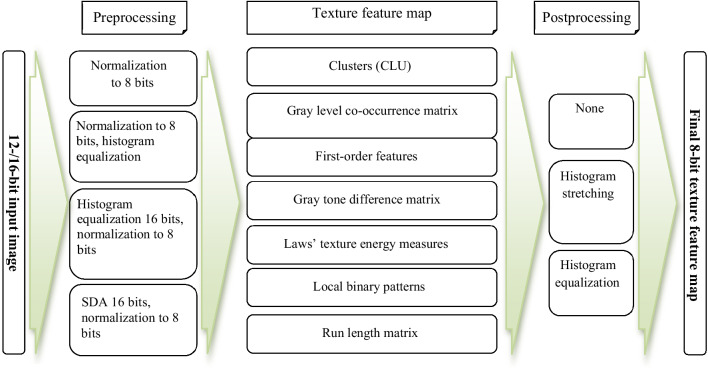
All possible entry data processing paths. *SDA* statistical dominance algorithm, *CLU* k-means clustering

For the obtained DIR images, texture feature maps were constructed using the above-described preprocessing techniques, analysis methods, and post-processing approaches (their possible combinations are shown in Fig. 2).

As discussed in the previous sections, the utilized texture operators have various parameters. For the LBP method, it is the radius, and for CLU, the number of searched clusters and related number of colors in the resulting image are taken into account. After determining the statistical features of GLCM, FOF, GTDM, and RLM, the corresponding texture feature maps are calculated (see Table 1).

The parameters describing the areas of the analyzed neighborhoods should also be determined. For computation of texture feature maps, the moving window resolution was 21 × 21 pixels (as mentioned previously). For the LBP function, P = 8 was always used, while the radius varied from 5 to 20. Clustering was performed in the range of 10 to 50 clusters. When the images were preprocessed with the SDA, the domination threshold was set to zero to obtain results that were independent of the image quality or objects surface, and the radius was set to 20. Some of the discussed techniques may have additional parameters. The selected values are presented in the detailed descriptions of the corresponding methods.

## Results and Discussion

In this study, texture feature maps were calculated for the collected input DIR data. After considering all possible combinations of the preprocessing, texture transformation, and post-processing methods, 1200 output images were obtained per input image.

### Image processing observations

**Fig. 3:**
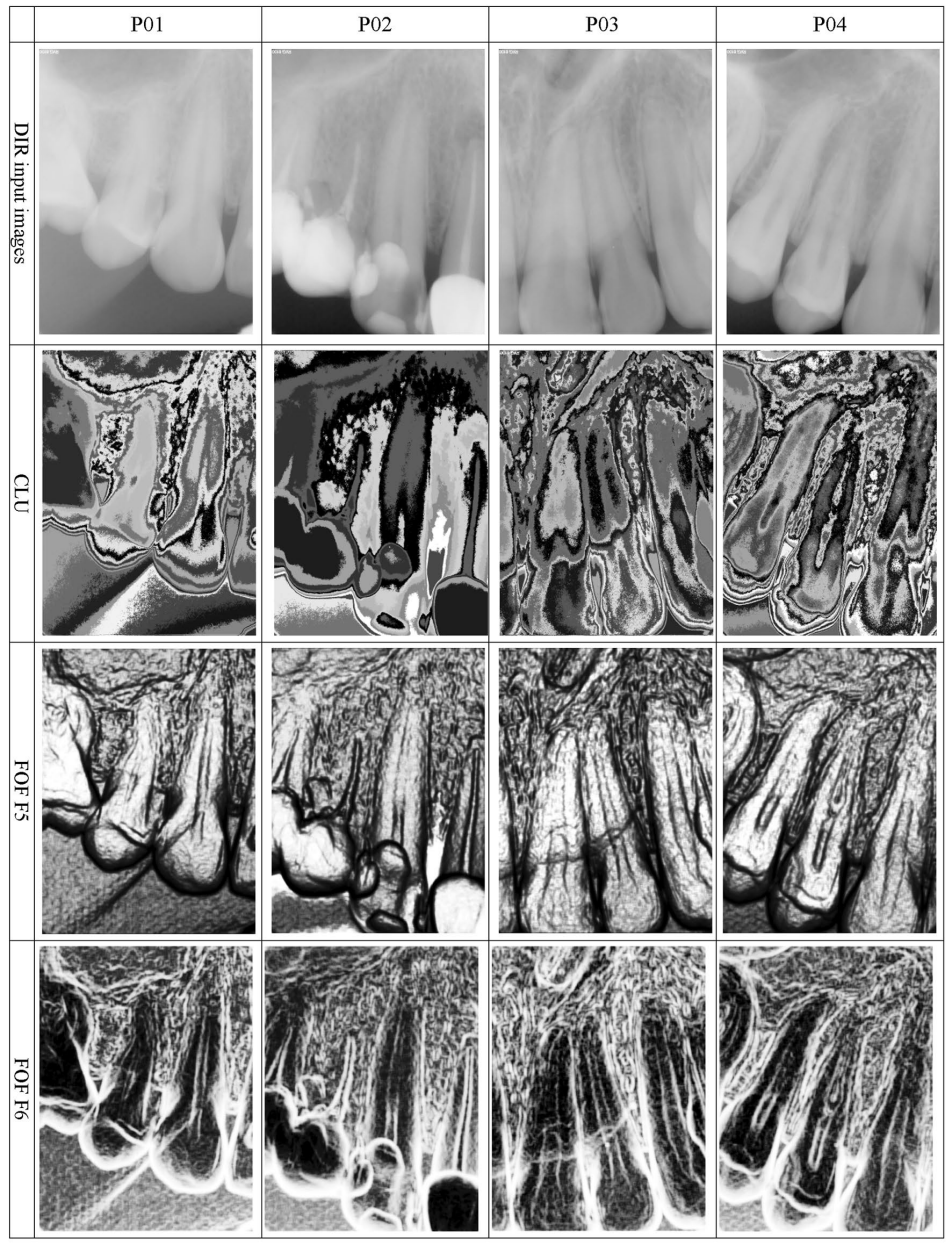
Input DIR images and their texture feature maps. *DIR* digital intraoral radiology

The CLU approach was applied to clearly define the borders of the regions affected by caries. In many cases, these regions were characterized by unique color sets. Notably, the selection of colors was random; however, its uniqueness on the entire image scale was important (see Fig. 3, second row).

The LBP method, in which preprocessing was applied to the original data points transformed by RAW/HEQ at *R* = 15, outlined the region of caries very clearly. In future studies, straightforward application of LBP to the input data with a resolution of 16 bits will be considered.

The texture feature maps produced by applying the FOF method to the original data set revealed that the most useful information was provided by the energy measure (F5) (because of its shape, it was called a pseudo-three-dimensional view), which is shown in the third row of Fig. 3. Another valuable parameter for further analysis is entropy (F6) (Fig. 3, last row), which outlines various objects but visualizes the caries-affected regions as consistent, characteristic areas. Good performance was also demonstrated by the HSTR and HEQ post-processing techniques, which clearly marked the regions of demineralization. The image quality of the texture feature maps obtained for energy and entropy (F5 and F6) was similar to that of the images produced by other methods; thus, considering the simplicity of their computations, they were used as the reference data. Interestingly, when the SDA was applied during the preprocessing stage, the texture feature maps obtained for energy and entropy (F5 and F6) were swapped, which was observed in all simulations.

**Fig. 4:**
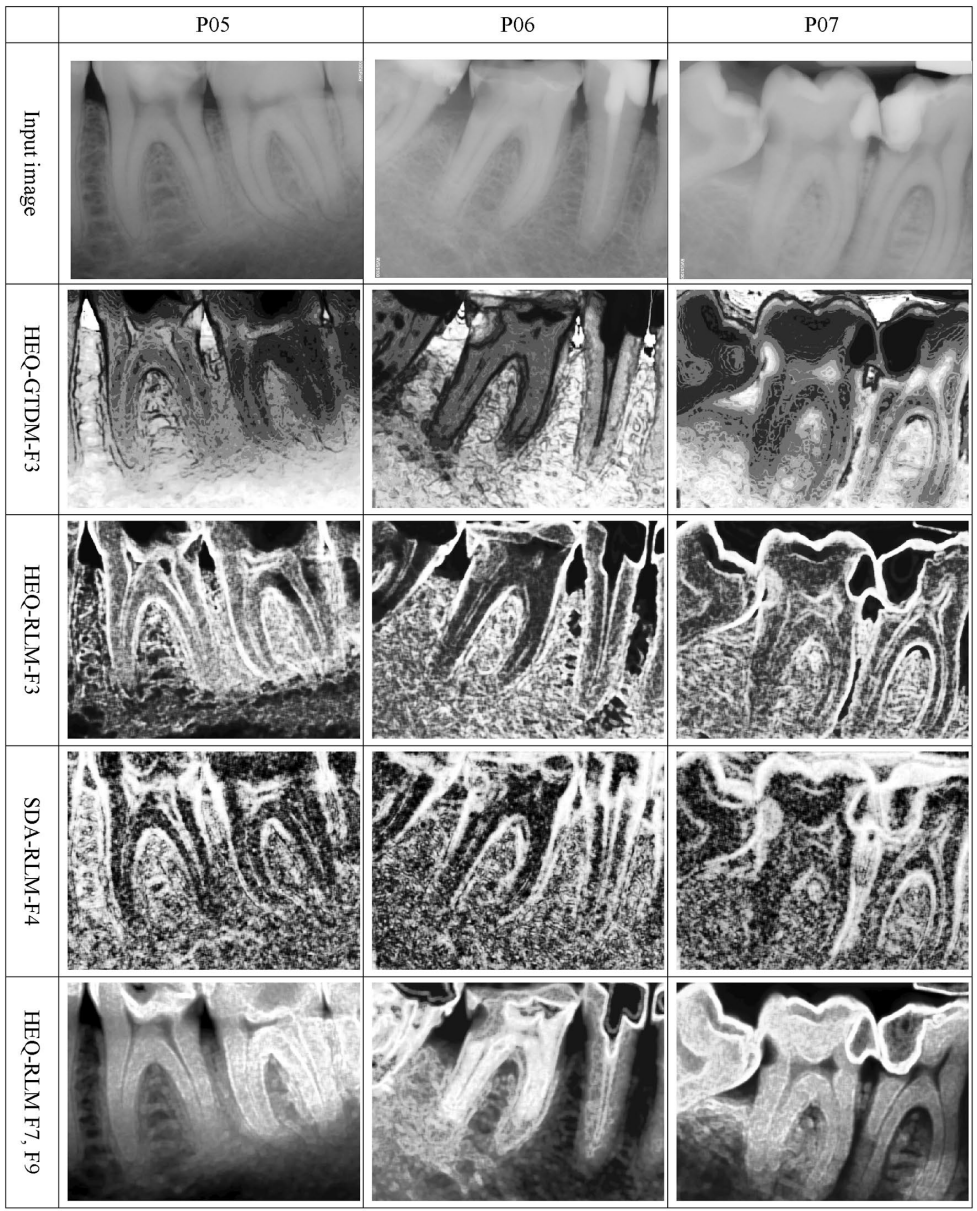
Texture feature maps for sample DIR images. DIR, digital intraoral radiology

For the original data, the texture feature map of coarseness obtained using the GTDM technique (F1) and post-processed with either HSTR or HEQ produced images similar to those obtained for FOF entropy (F6), but with a smaller number of details; therefore, their regions were less visible. Similarly, for the same input data, the GTDM busyness textural feature map (F3), which was obtained using the same post-processing method, contained images reflecting FOF energy (F5) with fewer details. A possible solution to this problem, which leads to a different data representation, may be the application of GTDM busyness (F3) to the images preprocessed and post-processed by HEQ. The obtained visual representation of various regions may show caries-affected areas, as shown in Fig. 4.

When the SDA was used during the preprocessing stage, the texture feature maps obtained for GTDM coarseness and texture strength (F1 and F5) were similar to those computed for FOF entropy (F6), while for GTDM contrast and busyness (F2 and F3), the corresponding maps strongly resembled those of FOF energy (F5). However, their image quality was also lower, which limited the clinical application of GTDM as compared with the outputs generated by the FOF method. Notably, computation of the FOF features proceeded much faster than that of the GTDM features. Moreover, the texture feature maps obtained for GTDM coarseness (F1) without preprocessing or application of the SDA were nearly identical.

Evaluation of the GLCM method did not produce any essential results for the DIR images. When the original images were processed, the resulting texture feature maps were blurred and not very detailed. Application of HEQ to the selected features during the preprocessing stage noticeably improved the information quality. However, this change was not significant enough to produce new frontiers for DIR image analysis. When the SDA was used at the initial stage, the obtained contrast (F2), difference variance (F10), and difference entropy (F11) features resulted in a texture feature map reflecting FOF F5, while the energy (F1) and inverse difference moment (F5) reflected the FOF entropy (F6).

By taking the images generated by the LAWS transformation of the original data into account, any information can be obtained from the resulting textural feature maps, because they contained mostly noise. The application of the HEQ procedure at the preprocessing stage improves the utility, especially for the F1 feature. The generated texture feature maps reflect those obtained for RLM gray level nonuniformity (F3) and preprocessed with HEQ, but they are more pronounced. When the SDA was applied at the initial stage, most of the results were similar to those obtained for FOF energy (F5); however, their noise level was higher. In general, adding the post-processing stage with HEQ significantly improved the quality of the produced maps.

No significant output was produced for the texture feature map derived from the RLM calculated for the original data. Some results may be described as a set of iso-lines, but the caries-affected area was not clearly visible. The application of HEQ at the initial stage of the RLM gray level nonuniformity (F3) computations yielded interesting data, while the results obtained with SDA preprocessing reflected FOF energy (F5), but with fewer details; this rendered them useless in clinical research. The absence of a preprocessing stage or using HEQ at the initial stage during the RLM F4 computation did not produce any important findings. When the SDA was used for preprocessing and HEQ was applied at the post-processing stage, resemblance to FOF entropy (F6) was observed with fewer details in the caries-affected area because of the selected post-processing method. For the RLM high gray level run emphasis and short run high gray level run emphasis features (F7 and F9) and HEQ preprocessing, the obtained texture feature maps represented the tooth roots and periapical areas in an interesting manner. However, these methods are not applicable to caries detection. Some of these maps are shown in the examples, as presented in Fig. 4.

### Clinical usability of texture maps and their interpretation

Compared with the DIR images, a significant increase in the tissue contrast between the caries-affected regions and mineralized tissue was observed when the CLU method was used. The former were highlighted as areas with different color intensities. The image obtained by clustering the input data indirectly reflects changes in radiodensity, and these changes are precisely expressed by the areas of different colors. The margins of the change are also well demarcated as variations in the color of the normal bone, and the affected regions are clearly visible. Therefore, this method can be used to detect caries and evaluate related areas.

The output of the LBP transformation procedure allows exact determination of the caries-affected areas, where the high tissue contrast between the normal and demineralized regions is highlighted. The margins of the lesions are well demarcated, but their definition (sharpness) is not as good as that in the FOF texture feature maps and images obtained by the clustering method. The usefulness of LBP is questionable, because the caries localization performed using these images produces no major benefits compared with the procedure conducted using DIR images and must be supported by other tissue texture feature maps (such as FOF ones).

**Fig. 5:**
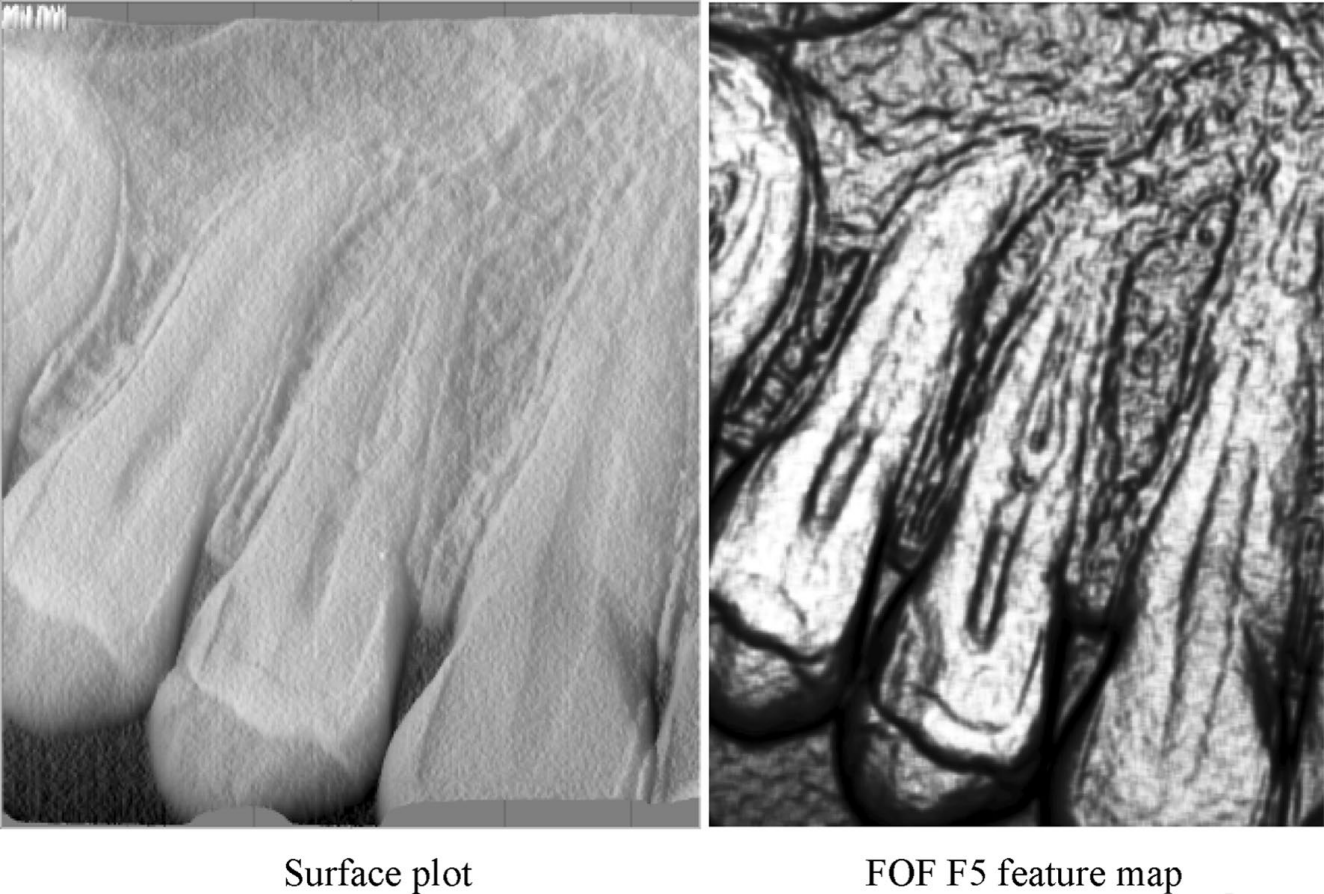
Comparison of a surface plot and an FOF F5 feature map

The texture feature map representing the variance of FOF energy (F5) was highly applicable to caries lesion localization. The shape of the lesion margins was well demarcated by the highlighted areas of different mineralizations, which produced an excellent picture compared with the initial DIR images. Although this texture appears similar to the surface plot of the original image, it significantly enhances the region of interest, as shown in Fig. 5. This texture emphasized the differentiation of the degree of demineralization of the tooth tissues in the carious cavity. The pseudo-three-dimensional effect shows the different absorption of X-rays inside the cavity. After clinical evaluation, we found that the obtained FOF energy (F5) images were extremely useful for detection of the lesions caused by caries. Additionally, the FOF energy (F5) texture feature map remarkably enriched the entire picture with a special emphasis on the anatomical details.

Next, the FOF F6 map contained very sharp margins of the change emphasizing the contrast of the caries-affected area (the latter was highlighted as a bright region compared with the relatively dark surroundings). However, unlike the FOF energy (F5) texture feature map, the regions of different mineralization were not well delineated; this is a clear drawback of this approach. Both the energy and entropy maps (F5 and F6, respectively) are highly recommended for evaluation of the lesions produced by caries and determination of their degree of severity.

As noted in the technical overview of the obtained results, the medical findings also show that applying the GTDM F1 feature post-processed with HEQ or HSTR produces results similar to the FOF entropy (F6) image, but with fewer details in the lesion area and with less tissue contrast. Therefore, the effectiveness of caries detection was lower than that achieved using the FOF texture feature maps, leading to no clinical benefits.

The construction of a texture feature map by the application of HEQ at the initial stage and the LAWS F1 feature increased the contrast of the demineralization zone in a large number of cases; however, the outline of their margins was relatively poor. Therefore, the clinical usefulness of this texture map was negligible.

Finally, applying the RLM texture operator after the HEQ procedure resulted in useful pictures with highlighted caries-affected areas and their margins, but the reproducibility of this effect was very low.

The possibility of image processing supplied with the medical equipment should also be mentioned. These software implementations deliver many classic image processing techniques to enhance the input image. In 2002, a thorough analysis of many solutions was presented [53]. In most cases, those filters demand interactive parameter settings; therefore, the human influence of the final result is strong. In a very recent study, the influence of standard techniques such as contrast and luminance selection was investigated in the context of caries detection in standard DIR images, and no significant difference was noted [54]. Similarly, a study in 2004 showed that additional filters such as sharpness, zoom, and pseudocolor do not affect the detection of occlusal caries [55]. The sharpen, smooth, and emboss filters have been previously verified [56]. Only the emboss filter had some value for the detection of approximal carious lesions in posterior teeth, but the output images looked like a surface plot (Fig. 5a). The present study showed that texture feature maps have a greater impact on caries detection than does surface plot analysis. Interestingly, the authors of another report presented the impact of lossy compression on the presentation of demineralization zones [57], similar to the CLU shown in this article.

## Conclusions and future studies

The results of this study revealed that the CLU approach could be particularly useful in the visualization of caries-affected areas. Furthermore, FOF texture feature maps may be used to detect caries spots. In particular, FOF energy (F5) reflects different degrees of demineralization and can, therefore, assist in the analysis of decalcification of caries cavities. Moreover, FOF entropy (F6) depicts the caries-affected areas as bright spots on the tooth surface. The usefulness of these texture feature maps was confirmed by the clinical evaluation of teeth using the images obtained with the intraoral camera.

The GTDM, LAWS, and RLM methods demonstrated limited clinical usefulness, because they produced better outlined caries-affected areas (compared with those in the original DIR images), but with much fewer details than in the CLU and FOF texture feature maps. The effects observed for these textures were caused by the application of preprocessing techniques, such as the SDA and HEQ techniques.

Future work in this area will explore the dependence of the texture feature maps on their resolution, which may be important for the recognition of caries lesions obtained using different X-ray instruments.
